# Intradialytic hypotension and relationship with cognitive function and brain morphometry

**DOI:** 10.1093/ckj/sfaa070

**Published:** 2020-12-05

**Authors:** Santiago Cedeño, Manuel Desco, Yasser Aleman, Nicolás Macías, Alberto Fernández-Pena, Almudena Vega, Soraya Abad, Juan Manuel López-Gómez

**Affiliations:** 1 Department of Nephrology, Hospital General Universitario Gregorio Marañón, Madrid, Spain; 2 Instituto de Investigación Sanitaria Gregorio Marañón, Madrid, Spain

**Keywords:** cardiovascular disease, cognitive impairment, haemodialysis, intradialytic hypotension, white matter

## Abstract

**Background:**

The haemodynamic stress brought about by dialysis could justify the loss of structural and functional integrity of the central nervous system (CNS). The main objective of this study was to analyse the relationship between intradialytic hypotension (IDH) and cognitive function and brain morphometry.

**Methods:**

The cross-sectional KIDBRAIN study (Cohort Study of Morphological Changes of the Brain by MRI in Chronic Kidney Disease Patients) included 68 prevalent patients with no history of neurological disorders (cerebrovascular disease and cognitive impairment) undergoing haemodialysis (HD). We analysed 18 non-consecutive dialysis sessions (first three of each month over a 6-month period) and various definitions of IDH were recorded. Global cognitive function (GCF) was assessed using the Mini-Mental State Examination (MMSE) and parameters of structural integrity of the CNS were obtained using volume morphometry magnetic resonance imaging analysis [grey matter (GM), white matter (WM) and hippocampus).

**Results:**

A greater number of sessions with IDH were associated with less volume of WM (*r* = −0.359,P = 0.003) and hippocampus (*r* = −0.395, P = 0.001) independent of cardiovascular risk factors according to multivariable linear regression models (β = −0.198, P = 0.046 for WM; β = −0.253, P = 0.017 for hippocampus). The GCF by the MMSE was 27.3 ± 7.3.1 and was associated with WM volume (β = 0.403, P = 0.001) independent of GM and hippocampus volume. Symptomatic IDH was associated with GCF (*r* = −0.420, P < 0.001) in adjusted analysis (β = −0.339, P = 0.008).

**Conclusions:**

Even when asymptomatic, IDH is associated with a lower WM and hippocampus volume and reduced GCF in patients undergoing HD, thus suggesting greater vulnerability of the brain to the haemodynamic stress that may be generated by a dialysis session.

## INTRODUCTION

Renal disease induces pathophysiological processes that can affect the structural and functional integrity of the central nervous system (CNS) [1]. The brain–kidney relationship can be observed both in the setting of acute kidney injury and in chronic kidney disease (CKD) and is mediated by processes such as inflammation, oxidative stress and haemodynamic stress [1]. Given that the course of brain damage is subclinical and often asymptomatic in CKD, diagnosis is facilitated by specific tests, such as the Mini-Mental State Examination (MMSE) or magnetic resonance imaging (MRI) [1–3].

Intradialytic hypotension (IDH) is a common complication of haemodialysis (HD), with a prevalence ranging from 5 to 30% [4]. This large difference is related to the heterogeneity of published studies resulting from the different definitions of IDH [5]. However, in predictive terms, nadir definitions have proven able to predict fatal events [6].

Adequate cerebral perfusion is determined by blood pressure (BP) and by the self-regulating capacity of the brain, which is severely affected by endothelial dysfunction in CKD [7]. Adaptive mechanisms against a decrease in BP—the baroreceptor reflex and a poor cardiac response in the context of the stunned myocardium—are compromised in patients undergoing HD [7]. HD can induce a decrease in cerebral perfusion [8–10], a scenario that is identical to that of the stunned myocardium [7]. Indeed, the improvement in haemodynamic stability achieved through the use of a cold dialysate preserves white matter (WM) microstructure [10].

In this context, we evaluated the relationship between the many definitions of IDH and cognitive function and the structure of the brain in a cohort of prevalent patients with no history of neurological disease undergoing HD.

## MATERIALS AND METHODS

The KIDBRAIN study (Cohort Study of Morphological Changes of the Brain by MRI in CKD Patients, NCT02827253) is based on the hypothesis that IDH plays a key role in the development of morphological changes in the brain and that these changes have a negative influence on cognitive function. The data used in the present study are from a cross-sectional analysis from the KIDBRAIN study.

### Subjects

The KIDBRAIN study included 68 patients undergoing HD. The study was conducted following Good Clinical Practice guidelines and patient recruitment was initiated after approval from the ethics and research committees. The inclusion criteria were as follows: age 18–80 years; prevalent cases undergoing HD for at least 6 months; no hospitalizations, surgery or clinical events in the 3 months before initiation of the study; and signed informed consent. The exclusion criteria were as follows: refusal to participate or revocation of written consent, history of neurological disorders by clinical history and psychologist evaluation (epilepsy, dementia or cognitive impairment), history of ischaemic or haemorrhagic stroke by clinical history and imaging methods, contraindications for performing MRI by clinical history (e.g. claustrophobia and non-compatible prosthetic material), receiving psychotropic-active treatments or drugs neurologically active by clinical history (i.e. benzodiazepines, hypnotics and neuroleptics) or failed renal allograft during the last year by clinical history.

### Variables

Demographic and clinical variables included age, gender, aetiology of CKD, duration of dialysis, previous transplant, history of atherosclerotic cardiovascular disease (CVD; ischaemic heart disease, peripheral vascular disease, mesenteric ischaemia), non-atherosclerotic CVD (heart failure, arrhythmias), vascular access, presence of residual renal function (RRF; defined as diuresis >500 mL/24 h) and antihypertensive drugs [number and drug class (e.g. β-blockers, renin–angiotensin system inhibitors, calcium antagonists, diuretics)]. The laboratory parameters collected were those related to anaemia (haemoglobin), bone mineral metabolism (calcium, phosphorus, parathyroid hormone) and inflammation (high-sensitivity C-reactive protein). Laboratory parameters were measured using standardized methods (auto-analysers). Serum C-reactive protein was measured using latex-based turbidimetric immunoassay with a Hitachi analyser (Sigma-Aldrich, St Louis, MO, USA).

### HD

We analysed retrospectively 18 non-consecutive HD sessions within a 6-month period (first three sessions of each month) for all patients before acquisition of brain images by MRI. We then verified the number of sessions in which eight definitions of IDH were met. The dialysis modality [high-flux HD and online haemodiafiltration (OL-HDF)] and each of the parameters used were based on medical judgement and local protocols [blood flow rate (Qb), dialysis flow rate (Qd), dialysate temperature, conductivity and dialysate composition]. Data on the dialysis sessions were collected as follows: Qb, dialysate temperature, *K_t_/V* (sp*K_t_/V* by Daugirdas, from urea clearance measured byultraviolet (UV) light absorbance and urea distribution volume measured by bioimpedance), convective volume (litres/session), interdialytic weight gain (IDWG) and ultrafiltration ratio (mL/h/kg).

### Definitions of IDH

BP was monitored in accordance with established local protocols every 30 min using the automated system of the 5008 CorDiax monitor (Fresenius Mediacl Care, Bad Homburg, Germany). The eight definitions of IDH were minimum intradialytic systolic BP (SBP) <90 mmHg (Nadir90), minimum intradialytic SBP <100 mmHg (Nadir100), decrease in SBP >30 mmHg with no associated symptoms (Fall30), decrease in SBP >20 mmHg with no associated symptoms (Fall20), Fall20Nadir90, Fall30Nadir90, decrease in SBP >20 mmHg with associated symptoms (dizziness, angina or any other symptom that can be attributed to arterial hypotension) as defined by the National Kidney Foundation’s Kidney Disease Outcomes Quality Initiative (KDOQI) or any decrease in SBP requiring the intervention of healthcare personnel (e.g. reduction in ultrafiltration and administration of saline) and end of the session(HEMO). We verified the number of sessions in which each definition of IDH was found. An IDH event was defined as positive if it was found in at least 25% of the 18 sessions analysed for each patient.

### Assessment of global cognitive function (GCF)

GCF was assessed using the Spanish version of the MMSE. Patients were assessed prior to commencing dialysis sessions in a quiet environment and always by the same examiner. We did not use an adjusted MMSE by level of education or age.

### Acquisition and processing of images

All subjects were scanned on the same Philips Achieva 1.5T MRI scanner (Philips Medical Systems, Best, The Netherlands) in the interdialysis period to minimize the influence of volume changes (on the no-dialysis day). For each patient, MRI was performed following a 6-month period of documentation of dialysis features (at the end of the 18 non-consecutive HD sessions observational period). The MRI protocol included the acquisition of T1-weighted, T2-weighted and diffusion-weighted images (DWIs). For the T1-weighted 3-dimensional magnetization-prepared rapid gradient echo imaging the following parameters were used: repetition time (TR) 25 ms, echo time (TE) 9.2 ms, matrix size 256 × 256 × 175, flip angle 30° and voxel size 0.9 × 0.9 × 1 mm^3^. The acquisition parameters for the T2-weighted turbo spin echo were TR 2800 ms, TE 280 ms, matrix size 256 × 256 × 175, flip angle 30°, voxel size 0.9 × 0.9 × 1 mm^3^. DWI data were acquired using a single-shot spin echo planar imaging sequence and acquisition parameters were an axial imaging plane TE 68 ms, TR = 11.886 ms, flip angle 90°, echo planar factor 77, number of slices 60, interslice gap 0 mm isometric voxel size 2.0 ×  2.0 ×  2.0 mm^3^ and acquisition matrix 128 × 128. A single non-DWI (B0) and 31 DWIs were acquired for a bβ of 1000 s/mm^2^. The volumes of grey matter (GM) and WM were adjusted for the total brain volume.

T1-weighted images were processed using FreeSurfer (http://surfer.nmr.mgh.harvard.edu/fswiki/recon-all) [11, 12] to extract brain measures of volume, cortical thickness and surface area and calculate their mean value for each of the 82 regions of the Desikan atlas. Lobar and hemispheric values were also calculated by averaging (cortical thickness) or adding (surface area and volume) the regions belonging to the same lobe or hemisphere.

DWIs were processed using the software packageFSL version 5.0 (FMRIB Software Library, FMRIB, Oxford, UK) [13]. Eddy currents and motion artefacts were corrected using the eddy currents correction routine implemented in FSL. FSL was used to fit the tensors and compute the fractional anisotropy, mean diffusivity, axial diffusivity and radial diffusivity maps.

We used standard processing pipelines for the anatomical and diffusion images, except for the calculation of the brain mask. One of the critical steps of image processing is to compute a more accurate mask of the brain. We noticed that the brain masks obtained with the FreeSurfer and FSL software were not always precise in our sample. Therefore we replaced them with a more accurate brain mask, which was computed with the Voxel-Based Morphometry version 8 toolbox (available at http://dbm.neuro.uni-jena.de/vbm).

### Statistical analysis

Continuous variables were tested for normality using the Kolmogorov–Smirnov test. Continuous variables are expressed as mean [standard deviation (SD)] or as median [interquartile range (IQR)] for non-normally distributed variables. A comparison between normally and non-normally distributed variables was performed using Pearson’s correlation coefficient (R) or Spearman’s rank correlation coefficient. Parametric data were compared using the *t*-test or analysis of variance. Categorical variables were expressed as percentages and compared using the chi-squared test. Multivariable linear regression analysis (‘Enter’ method) was used to evaluate independent associations. Statistical significance was set at P < 0.05, with a 95% confidence interval (CI). Data were analysed using SPSS version 20.0 (IBM, Armonk, NY, USA).

## RESULTS

### Baseline characteristics

Table 1 shows the demographic data, clinical features and laboratory data of the 68 study participants.

**Table 1.** Baseline characteristics

Characteristics	Mean ± SD N. percentage
Age (years), mean ± SD	58.6 ± 14.7
Gender (male/female), %	64.7/32.3
Years of education, mean ± SD	8.9 ± 1.2
DM, *n* (%)	16 (23.5)
Arteriovenous fistulae, *n* (%)	59 (86.8)
History of CVD, *n* (%)	34 (50)
Dialysis vintage (months), median (IQR)	46.5 (24–104)
RRF, *n* (%)^a^	18 (26.5)
OL-HDF, *n* (%)	50 (73.5)
Patients on antihypertensive drugs, *n* (%)	43 (63.2)
Number of antihypertensive drugs, mean ± SD	0.6 ± 0.4
RAS inhibitors, *n* (%)	18 (26.5)
β-blockers, *n* (%)	24 (35.3)
Diuretics, *n* (%)	8 (11.8)
Non-dihydropyridine calcium channel blocker, *n* (%)	23 (33.8)
BMI (kg/m^2^) mean ± SD	24.9 ± 6.4
C-reactive protein (mg/dL), mean ± SD	0.8 ± 1.4
Serum albumin (g/dL), mean ± SD	3.7 ± 0.4
HDL cholesterol (mg/dL), mean ± SD	46.2 ± 15.8

a RRF defined as diuresis >500 mL/24 h.

RAS, renin–angiotensin system; BMI, body mass index; HDL, high-density lipoprotein.

### Characteristics of HD

We analysed 1224 dialysis sessions (18 sessions per patient). The main characteristics are summarized in Table 2.

**Table 2.** Characteristics of HD

Dialysis features	Mean ± SD
SBP pre-dialysis (mmHg)	139.2 ± 16.8
SBP post-dialysis (mmHg)	128.9 ± 17.2
DBP pre-dialysis (mmHg)	71.2 ± 11.8
DBP post-dialysis (mmHg)	69.9 ± 10.8
Dry weight (kg)	68.2 ± 10.5
IDWG (g)	1873.1 ± 685
IDWG (percentage of dry weight)	1.8 ± 0.6
Ultrafiltration (mL)	1866 ± 717
*K_t_/V*	2.0 ± 0.5
Convective volume in OL-HDF patients (L)	27.6 ± 4.1
Ultrafiltration ratio (mL/h/kg)	7.4 ± 2.9
Number of sessions with ultrafiltration ratio >12 mL/h/kg	1.8 ± 3.1
Dialysate temperature (°C)	35.6 ± 0.4
Urea reduction ratio (%)	81.9 ± 6.7
β2-microglobulin reduction ratio (%)	79.1 ± 8.1

Values are expressed as mean ± SD.

K*_t_/V*: sp*K_t_/V* by Daugirdas. From urea clearance measured by UV absorbance and urea distribution volume measured by bioimpedance.

Ultrafiltration ratio: obtained from the formula [IDWG (mL/treatment time (h)]/post-dialysis weight (kg).

Urea reduction ratio and β2-microglobulin reduction ratio are obtained from the formula [(Solute)_pre _− (Solute)_post_/(Solute)_pre_] × 100.

DBP, diastolic blood pressure.

### IDH

The most prevalent definitions of hypotension were Fall20 and Fall30, while the least frequent wereKDOQI and HEMO. Table 3 shows the distribution of each definition of IDH in the dialysis sessions analysed. Older age, diabetes mellitus (DM), longer dialysis vintage, lower SBP and higher IDWG were associated with a higher hypotension rate. We found no relationship between rates of IDH and dialysis modality or presence of RRF except for Nadir100 and KDOQI definitions (a higher rate of IDH in patients without RRF) (see Supplementary data).

**Table 3.** Definitions of IDH

IDH definition	Mean ± SD	*n*/1000 dialysis sessions	>25% of dialysis sessions^a^ (%)
Nadir90	3.1 ± 3.8	172.3	27.9
Nadir100	6.1 ± 5.7	339.8	50
Fall20	11.0 ± 4.2	611.1	95.6
Fall30	7.9 ± 4.8	441.1	73.5
Fall20Nadir90	2.6 ± 3.4	145.4	23.5
Fall30Nadir90	2.1 ± 3.2	120.1	16.2
KDOQI	1.7 ± 2.0	98.0	10.3
HEMO	2.3 ± 2.5	129.9	20.6

Each definition of IDH is expressed as mean ± SD of dialysis sessions (number of dialysis sessions with hypotension) and as the number of episodes of hypotension per 1000 dialysis sessions.

a Percentage of patients with IDH definition in >25% of dialysis sessions.

### IDH and brain morphometry

**FIGURE 1:**
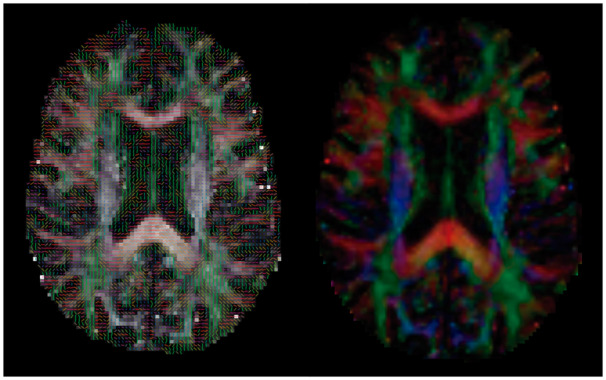
Fractional anisotropy. The picture on the right shows a slice of the fractional anisotropy map of a single subject with an overlay of the first eigenvector. The picture on the left represents a slice of the coloured fractional anisotropy map for the same subject. The colour indicates the direction of the fibres in each voxel (red: left–right; green: anterior–posterior; blue: superior–inferior).

A greater number of sessions with hypotension were associated with a lower volume of WM and hippocampus. In addition, worse fractional anisotropy was associated with a greater number of sessions with asymptomatic IDH (see Figure 1). We found no relationship between GM and IDH. Table 4 summarizes the relationship between the different definitions of IDH and the structure of the CNS.

**Table 4.** Relationship between IDH and brain morphometry

IDH definition	GM volume (cortical)		GM volume (cortical and subcortical)		WM volume		Hippocampus volume		Fractional anisotropy	
	R	P	R	P	R	P	R	P	R	P
Nadir90	−0.157	0.2	−0.159	0.1	−0.239	0.05	−0.282	0.02	−0.122	0.3
Nadir100	−0.091	0.4	−0.098	0.4	−0.207	0.09	−0.229	0.06	−0.131	0.2
Fall20	−0.164	0.1	−0.182	0.1	−0.359	0.003	−0.395	0.001	−0.337	0.005
Fall20Nadir90	−0.113	0.3	−0.116	0.3	−0.254	0.036	−0.230	0.059	−0.101	0.4
Fall30	−0.122	0.3	−0.133	0.2	−0.284	0.019	−0.344	0.004	−0.272	0.02
Fall30Nadir90	−0.065	0.5	−0.067	0.5	−0.184	0.1	−0.219	0.07	−0.129	0.3
KDOQI	−0.112	0.3	−0.106	0.3	0.055	0.6	−0.286	0.018	−0.048	0.6
HEMO	−0.012	0.9	−0.012	0.9	−0.128	0.2	−0.321	0.008	−0.073	0.5

The volumes of GM and WM were adjusted for the total brain volume.

The multivariable linear regression models revealed an independent relationship between a higher rate of hypotension and lower volumes of WM and hippocampus. Other factors associated with these findings included advanced age, DM and RRF (Tables 5 and 6).

**Table 5.** Multivariate linear regression analysis for WM volume

Model 1(WM)	Non-standardized coefficients		β-coefficient	*t*	P-value
	β	typical error			
Constant	0.324	0.034		9.429	<0.001
Age (years)	−0.001	0.00	−0.370	−2.797	0.007
DM	−0.014	0.006	−0.250	−2.292	0.026
IDH^a^	−0.001	0.001	−0.221	−2.020	0.048
RRF	0.009	0.007	0.150	1.225	0.222
SBP pre-dialysis	0.00	0.00	−0.074	−0.693	0.491
History of CVD	0.00	0.006	0.009	0.076	0.940
Dialysis vintage	0.00	0.00	−0.018	−0.157	0.876
BMI	0.00	0.001	0.066	0.555	0.581
C-reactive protein	0.002	0.002	0.112	1.017	0.313
Serum albumin	0.004	0.007	0.078	0.682	0.498
Model 2(WM)	Non-standardized coefficients		β-coefficient	*t*	P-value
	β	typical error			
Constant	0.333	0.011		30.467	<0.001
Age (years)	−0.001	0.000	−0.376	−3.690	<0.001
DM	−0.015	0.006	−0.260	−2.715	0.009
IDH^a^	−0.001	0.001	−0.198	−2.034	0.046
RRF	0.012	0.005	0.208	2.140	0.036

Model 1: multivariable linear regression analysis for white matter volume. Adjusted R^2^: 0.376. Model 2: multivariable linear regression analysis for white matter volume. Adjusted R^2^: 0.424. ^a^IDH, intradialytic hypotension; Fall20 definition. RRF: residual renal function, defined as diuresis >500 ml/day, SBP: systolic blood pressure, CVD: cardiovascular disease, BMI: body mass index, WM: white matter volume. p: statistical significance.

**Table 6.** **Multivariate linear regression**  **analysis for hippocampus volume**

Model 1(HPC)	Non-standardized coefficients		β-Coefficient	*t*	P-value
	β	typical error			
Constant	5646.258	1589.854		3.551	0.001
Age (years)	−18.385	10.366	−0.235	−1.774	0.082
DM	−463.018	292.720	−0.173	−1.582	0.119
IDH	−80.109	29.866	−0.293	−2.682	0.010
RRF	316.875	322.360	0.121	0.983	0.330
SBP pre-dialysis	0.835	7.314	0.012	0.114	0.910
History of CVD	−227.769	280.997	−0.099	−0.811	0.421
Dialysis vintage	1.504	1.650	0.102	0.911	0.366
BMI	5.049	25.392	0.024	0.199	0.843
C-reactive protein	22.688	89.555	0.028	0.253	0.801
Serum albumin	747.739	305.034	0.282	2.451	0.017
Model 2(HPC)	Non-standardized coefficients		β-coefficient	*t*	P-value
	β	typical error			
Constant	9420.49	493.08		19.105	<0.001
Age (years)	−31.371	8.143	−0.405	−3.852	<0.001
DM	−560.35	269.66	−0.209	−2.078	0.042
IDH	−68.782	28.041	−0.253	−2.453	0.017

Model 1: multivariable linear regression analysis for hippocampus volume. Adjusted R2: 0.377. Model 2: multivariable linear regression analysis for hippocampus volume. Adjusted R2: 0.350. IDH: intradialytic hypotension. *Fall20 definition. RRF: residual renal function, defined as diuresis >500 ml/day, SBP: systolic blood pressure, CVD: cardiovascular disease, BMI: body mass index, HPC: hippocampus volume. p: statistical significance.

### GCF

The GCF score by the MMSE was 27.3 ± 3.1 points. This was associated with WM (β = 0.403, P = 0.001) independent of GM volume at the cortical and subcortical levels and with the hippocampus (Table 7). Symptomatic IDH was associated with GCF (KDOQI: *r* = –0.420, P < 0.001; HEMO: *r* = –0.369, P = 0.005) independent of cardiovascular risk factors according to the multivariable linear regression models (KDOQI: β = –0.339, P= 0.008; HEMO: β = –0.305, P = 0.018). Table 8 summarizes the other factors that were associated with GCF.

**Table 7.** GCF and brain morphometry

Structure of CNS	Unadjusted analysis		Adjusted analysis	
	*r*	P-value	β	P-value
GM volume (cortical)	0.330	0.006	0.074	0.5
GM volume (cortical + subcortical)	0.356	0.003	0.102	0.4
WM volume	0.473	<0.001	0.403	0.001
Fractional anisotropy	0.354	0.003	0.233	0.039
Hippocampus volume	0.541	<0.001	0.184	0.1

*r*: Pearson's correlation coefficient. Note: GCF was estimated by the MMSE.

**Table 8.** Factors associated with global cognitive function

Related-factors	Unadjusted analysis	Adjusted analysis	
Age, years	*r* = 0.529, P < 0.001	^a^ ^b^β	P-value
		−0.320	0.031
		−0.337	0.027
DM	Diabetes [*n* = 16 (23.5%)]: MMSE: 25.0 ± 4.2/no diabetes [*n* = 52(76.4%)]: MMSE: 27.7 ± 2.2 (P = 0.023)	−0.021	0.8
		−0.018	0.8
CVD	CVD [*n* = 34 (50%)]: MMSE: 26.1 ± 3.3/no CVD [*n* = 34 (50%)]: MMSE: 28.0 ± 2.4 (P = 0.011)	−0.041	0.7
		−0.57	0.6
HD modality (OL-HDF ref.)	OL-HDF [*n* = 50 (73.5%)]: MMSE: 27.9 ± 2.2/high-flux HD [*n* = 18 (26.4%)]: MMSE: 24.8 ± 3.7 (P = 0.003)	0.310	0.02
		0.325	0.02
β2-microglobulin reduction ratio	*r* = 0.319, P = 0.035	0.072	0.5
		0.037	0.7
IDH	KDOQI: *r* = −0.420, P < 0.001; HEMO: *r* = −0.369, P = 0.005	−0.339	0.008
		−0.305	0.018

a Model 1: multivariable linear regression analysis for global cognitive function. Adjusted *R*^2^ = 0.421. Includes KDOQI definition of hypotension.

b Model 2: multivariable linear regression analysis for global cognitive function. Adjusted *R*^2^ = 0.398. Includes HEMO hypotension definition.

## DISCUSSION

In our cohort of prevalent patients on HD with no history of neurological disease, we demonstrated an independent association between the number of sessions with arterial hypotension and WM and hippocampus volume.

CKD is associated with a high prevalence of subclinical structural involvement of the CNS in the form of cerebral atrophy and ischaemic WM changes. The diagnosis is usually an incidental finding in imaging tests, such as MRI [14–16]. A greater degree of brain atrophy has been identified in HD patients, with lower frontal and temporal lobe volumes [17]. The development of imaging tests has increased our knowledge of the structure of the CNS exponentially [18, 19], with a considerable contribution from the capacity of MRI for the diagnosis of apparent cerebrovascular damage [17]. Voxel-based morphometry is probably the most accurate structural MRI technique for describing brain volume by regions and differences in tissue concentration [20].

Although many mechanisms can explain the greater prevalence of cerebral atrophy in CKD [21, 22], one of the most important seems to be the haemodynamic stress brought on by dialysis [7–10]. Polinder-Bos *et al.* [8] and Findlay *et al.* [9] recently used positron emission tomography–computed tomography and Doppler ultrasound, respectively, to demonstrate a significant decrease in cerebral blood flow during dialysis. In this regard, the ultrafiltration rate plays a critical role [8]; for example, an ultrafiltration rate of ~2200 mL/dialysis was associated with a decrease in cerebral blood flow, although isovolumetric dialysis had no effect on cerebral blood flow [23].

We used eight different definitions of IDH and found high interpatient variability, similar to that reported elsewhere [24]. If we consider the definition of hypotension that includes associated symptoms (KDOQI), then 39% of the patients in the study had no sessions with symptomatic IDH. In contrast, in 60% of patients at least one episode of symptomatic IDH was recorded. A similar profile occurred with the remaining definitions, especially the nadir definitions. Although Fall20 was a ‘constant phenomenon’, it was not recorded in one patient. In general terms, the asymptomatic definitions of IDH, such as Fall20, Fall30, Nadir90 and Nadir100 were significantly more frequent than KDOQI or HEMO (symptomatic definitions). The prevalence of hypotension in the study ranged from 10.3% (KDOQI) to 20.6% (HEMO), although the nadir definitions ranged from 27.9% for Nadir90 to 50% for Nadir100. These differences in the prevalence of hypotension were similar to those reported in other studies on IDH [4, 5].

The negative correlation with brain structure, particularly WM and the hippocampus, was only observed with the asymptomatic definitions of IDH. The number of sessions with a decrease of 20 mmHg in pre-dialysis SBP was an independent predictor of WM and hippocampus volume, thus magnifying the importance of the decrease in BP, irrespective of whether it is symptomatic or not. While these results do not demonstrate causality, IDH detection and brain MRI are closely aligned and that fact leads us to consider there is an association between them. Even though it is unlikely to exclude the possibility that brain MRI changes existed prior to the detected episodes of hypotension, this is the first study—to our knowledge—to evaluate the relationship between nadir definitions and the structure of the CNS. Flythe *et al.* [6] demonstrated the predictive power of fatal events of nadir definitions of IDH; our results show that the association of nadir definitions occurred only in the models with brain volumes not adjusted for total cerebral volume (data not shown).

The fact that more frequent and repeated less severe hypotensive episodes rather than less frequent but more severe IDH could have a more relevant role in producing brain damage is of great clinical significance. The definition of a cut-off point from which BP compromises tissue integrity is the subject of debate [7] and the ‘safe threshold’ for BP to minimize brain damage during a dialysis session remains unknown [4]. In their pilot study, MacEwen *et al.* [25] found a poor correlation between BP and cerebral ischaemia, probably because self-regulation of cerebral blood flow is a complex process that depends on factors other than BP. Tissue perfusion is expressed by the blood flow velocity/100 mL of tissue in the formula *Q* = *P*/*R* (where *Q* is blood flow, *P* is BP and *R* is resistance) [26]. For tissue hypoperfusion to occur, there must be either an absence of local vasodilator response or a significant decrease in BP that cannot be counteracted by vasoconstriction. Patients with CKD usually have microcirculation abnormalities (and therefore a worse adaptive vasodilator response) and a greater oxygen demand in certain organs owing to a structural alteration (e.g. hypertrophy of the left ventricle), which predisposes to tissue damage in the context of an IDH [26].

Based on our cohort results, we can say that one of the most important issues regarding IDH is the frequency of recurrent episodes leading to subclinical tissue hypoperfusion irrespective of the magnitude of BP decrease during a dialysis session. This should lead nephrologists and dialysis nurses to focus not only on symptomatic episodes, but also on apparently ‘benign asymptomatic episodes’.

An interesting aspect of our results is the positive association we found between RRF and WM volume, suggesting a ‘protective effect’ of RRF on brain structure in patients undergoing HD. These findings could be explained by two mechanisms: (i) a protective effect against the haemodynamic stress of RRF, thus necessitating a lower ultrafiltration rate, and (ii) the role that RRF would have in the clearance of toxins that are hardly eliminated by HD. We did not find a structural correlation with the ultrafiltration ratio when it was evaluated as a continuous variable and, according to the cut-off points proposed in the literature, as risk factors for cardiovascular morbidity and mortality [27–31].

Although the characteristics of the dialysis sessions and the aspects related to haemodynamic tolerability were similar between patients with and without RRF, the latter were 10 years younger, had a shorter dialysis vintage and less frequently had CVD. On the other hand, tubular secretion is a useful mechanism that enables the kidney to eliminate solutes with considerable affinity for proteins [32], and we cannot rule out the possibility that differences in brain structure are not related to the elimination of uremic toxins with limited clearance through HD. To our knowledge, no study has evaluated the role of RRF on brain structure, therefore more studies are needed to confirm these findings.

CKD is considered an independent risk factor for the onset and progress of cognitive impairment, with a prevalence ranging between 16 and 38% depending on the definition used, the tests used to evaluate the cognitive function and the study population [1–3, 33–37]. Although cognitive impairment can develop at any stage of CKD, the patients at greatest risk are those undergoing renal replacement therapy, either as HD or peritoneal dialysis [1–3, 33–35, 38, 39].

Recently the nature of themild cognitive impairment (MCI) in CKD patients has been characterized. This entity differs from the classic ‘uremic encephalopathy’, but may also be different from the MCI of the general population without CKD [40]. It is not well understood if MCI related to CKD represents only an ‘accelerated’ phenotype of MCI or if it is a specific entity of CKD, being a consequence of the accumulation of uremic toxins, imbalances in electrolytes, rapid shifts of volume and inflammation pathways, among others [36].

The typical cognitive impairment profile in CKD involves memory domains and executive functions [33, 34, 38, 39] that are seen as a disruption in the frontal and subcortical neuronal circuits [37]. Although the MMSE was used to evaluate cognitive function in more than half of published studies, it is not free of limitations. One such limitation is the lower capacity to evaluate executive functions [2, 3].

Our results for GCF are similar to those reported in the literature, with a mean of 27 points in the MMSE in patients undergoing HD [33, 38, 39]. Technological advances in imaging methods, particularly in MRI, have enabled better characterization of cognitive impairment from a morphological point of view [41]. Chai *et al.* [21], Chang *et al.* [40], Prohovnik *et al.* [42] and Zhang *et al.* [43] have shown a correlation between GCF and the volume of GM and the hippocampus [21, 40, 42, 43]. In our cohort, we found a correlation between GCF by MMSE and WM independent of other structures, such as GM or the hippocampus. The complexity of tasks dependent on GCF requires coordination between different parts of the brain, and the structural integrity of the WM plays an essential role in this interconnection [44–46]. Other authors have also found WM to be vulnerable to haemodynamic instability during the dialysis session [10], and in our study, GCF was associated with symptomatic IDH.

When assessing GCF in patients undergoing HD, it is important to take into account the fluctuation in the score obtained in the different tests depending on the dialysis cycle [47, 48]. For this reason, we assessed GCF before dialysis in a quiet environment. Haemodynamic stress is one of the potential causes of these fluctuations in GCF, and although impairment may be transient with complete recovery, it can also have long-term repercussions [47, 48]. Consequently, hypoperfusion processes play a key role in the pathophysiology of cognitive impairment [33–37, 39, 47, 48].

One of the main limitations of the study is the cross-sectional analysis of the structure of the CNS, which limits our ability to demonstrate a causal relationship between the presence of IDH and morphological changes in the CNS. Although the sample size is similar to that used in other studies that evaluate brain structure based on voxel-based morphometry, we believe that a larger sample would confer greater statistical power. Nevertheless, one of the main strengths of our study is the use of voxel-based morphometry analysis of structural images obtained by MRI. In addition, the spectrum of definitions of IDH is broad, similar to that observed in routine clinical practice.

We can report two main findings: the ‘symptomatic’ definitions of hypotension were related to GCF, as evaluated by MMSE, and the ‘asymptomatic’ definitions of IDH were related to the structural integrity of the CNS (WM and hippocampus). These results support the hypothesis of the study, namely, that IDH may affect the structural and functional integrity of the CNS.

## SUPPLEMENTARY DATA

Supplementary data are available at ckj online.

## Supplementary Material

sfaa070_supplementary_data
