# Supplementary material for: Intradialytic hypotension and relationship with cognitive function and brain morphometry
Source: Clin Kidney J. 2020 Dec 5;14(4):1156–64. doi: 10.1093/ckj/sfaa070 (PMC8023187; doi:10.1093/ckj/sfaa070)
Supplement: sfaa070_supplementary_data [file sfaa070_supplementary_data.docx]

**SUPPLEMENTARY CONTENT:**

**Table 1. Baseline characteristics according to dialysis modality**


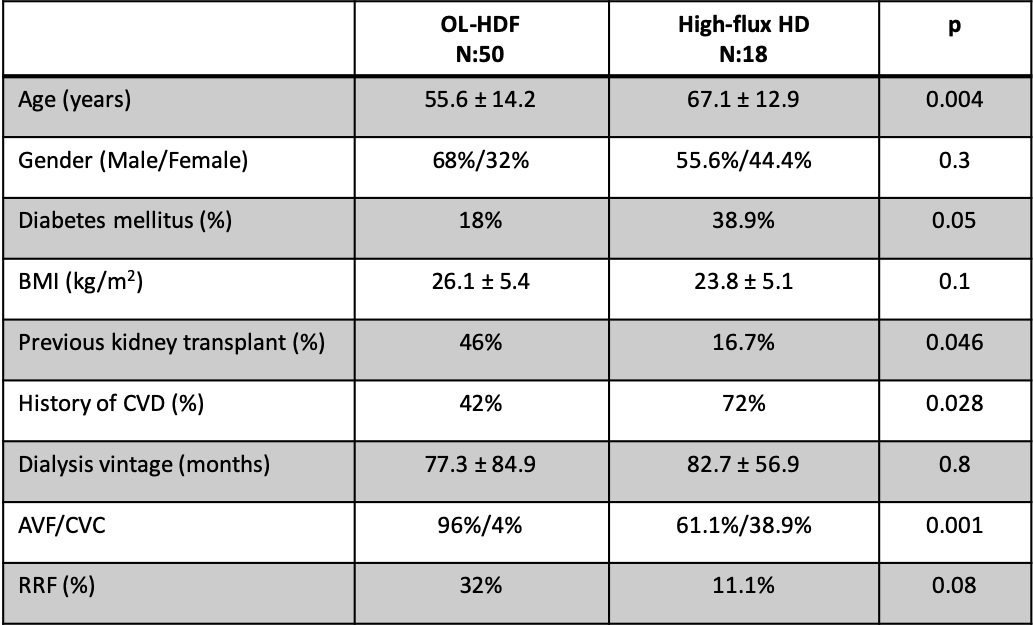


Quantitative variables are expressed as mean and SD. Qualitative variables are expressed as absolute values and percentages.

OL-HDF: on-line hemodiafiltration. BMI: body mass index. CVD: cardiovascular disease. AVF: arterio-venous fistula. CVC: central venous catheter. RRF: residual renal function.

**Table 2. IDH definitions and related factors**

**
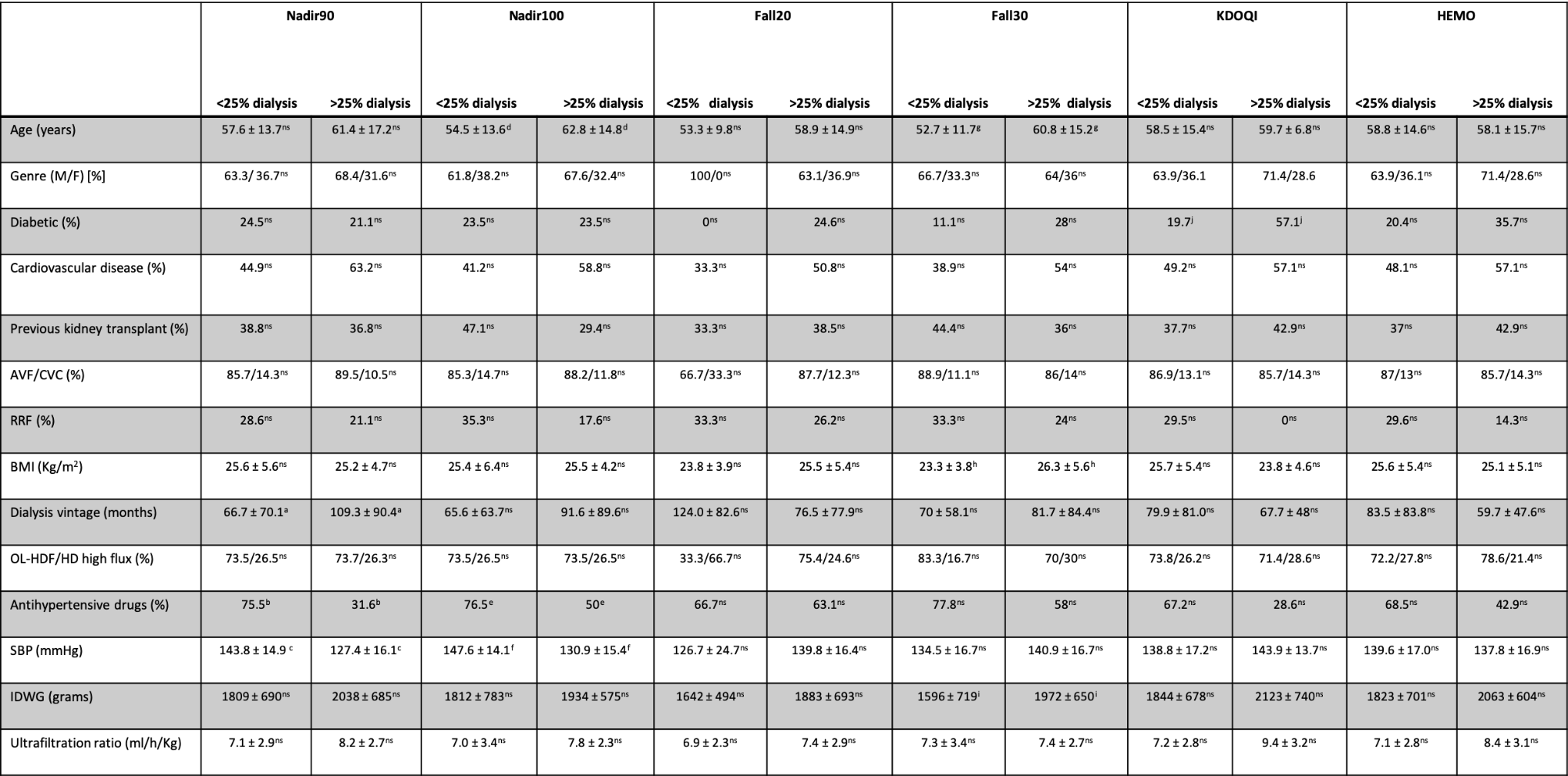
**

Quantitative variables are expressed as mean and SD. Qualitative variables are expressed as absolute values and percentages.

M: male. F: female. AVF: arterio-venous fistula. CVC: central venous catheter. RRF: residual renal function. BMI: body mass index. OL-HDF: On-Line Hemodiafiltration. SBP: systolic blood pressure pre-dialysis. IDWG: interdialytic weight gain.

a: p 0.043, b: p 0.002, c: p <0.001, d: p 0.019, e: p 0.024, f: p <0.001, g: p 0.044, h: p 0.044, i: p 0.044, j: p 0.048, ns: non-statistical significance.

**Table 3. IDH definitions according to dialysis modality**

**
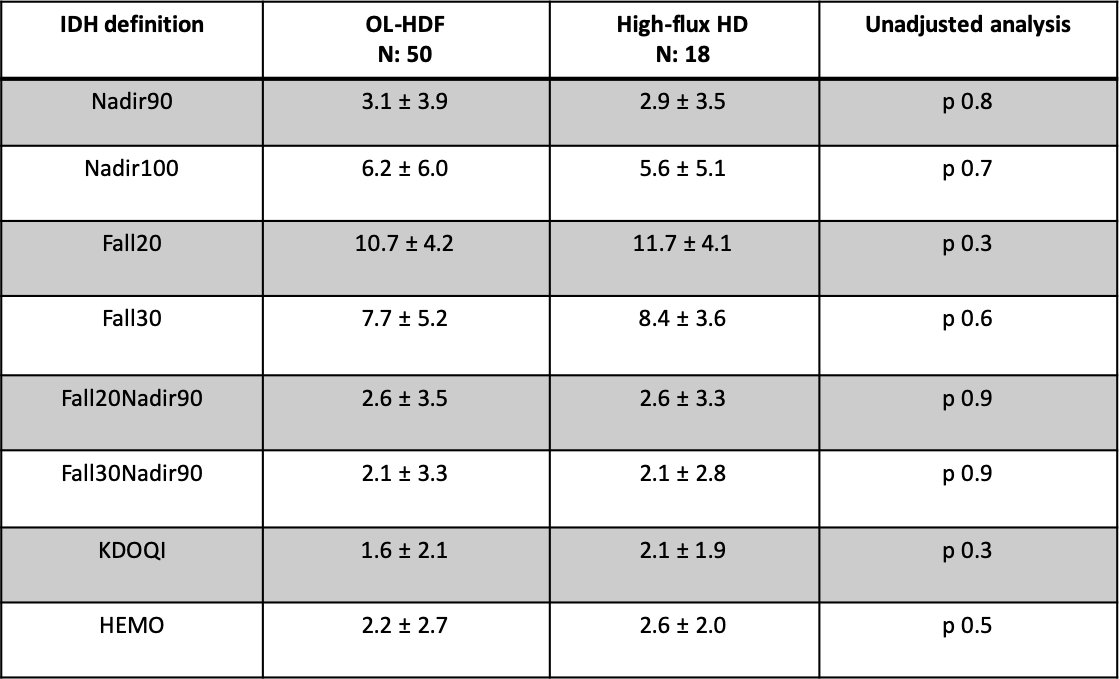
**

Quantitative variables are expressed as mean and SD.

IDH: intradialytic hypotension. OL-HDF: on-line hemodiafiltration. HD: hemodialysis.

**Table 4. IDH definitions according to residual renal function**

**
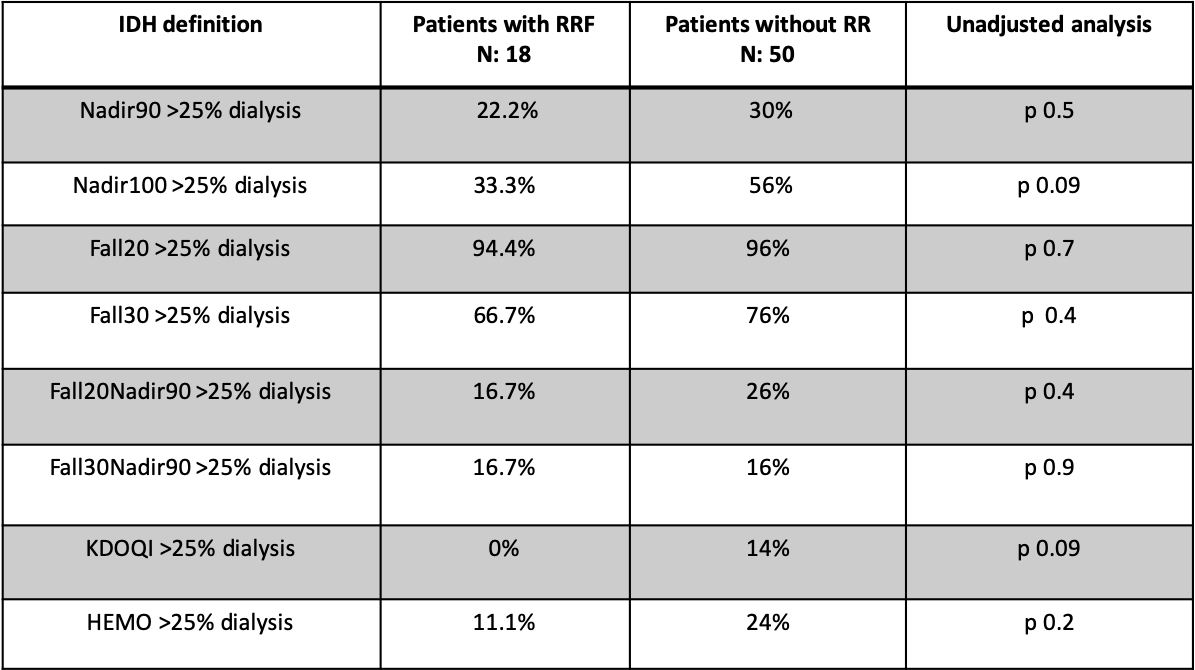
**

Percentage of patients with IDH definition in >25% dialysis.

IDH: intradialytic hypotension. RRF: residual renal function.
